# Genetic influences on prefrontal activation during a verbal fluency task in children: A twin study using near‐infrared spectroscopy

**DOI:** 10.1002/brb3.980

**Published:** 2018-04-24

**Authors:** Eisuke Sakakibara, Ryu Takizawa, Yuki Kawakubo, Hitoshi Kuwabara, Toshiaki Kono, Kasumi Hamada, Shiho Okuhata, Satoshi Eguchi, Ayaka Ishii‐Takahashi, Kiyoto Kasai

**Affiliations:** ^1^ Department of Neuropsychiatry Graduate School of Medicine The University of Tokyo Tokyo Japan; ^2^ Department of Clinical Psychology Graduate School of Education The University of Tokyo Tokyo Japan; ^3^ MRC Social, Genetic and Developmental Psychiatry Centre Institute of Psychiatry, Psychology and Neuroscience King's College London London UK; ^4^ Department of Child Neuropsychiatry Graduate School of Medicine The University of Tokyo Hospital Tokyo Japan; ^5^ Research Center for Child Mental Development Hamamatsu University School of Medicine Shizuoka Japan; ^6^ Department of Forensic Psychiatry National Center of Mental Health National Center of Neurology and Psychiatry Tokyo Japan; ^7^ The Department of Social Childhood Care and Education The Faculty of Health and Welfare Nayoro City University Hokkaido Japan; ^8^ Department of Electrical Engineering Graduate School of Engineering Kyoto University Kyoto Japan; ^9^ Section on Neurobehavioral Clinical Research, Social and Behavioral Research Branch National Human Genome Research Institute National Institutes of Health Bethesda MD USA

**Keywords:** children, heritability, near‐infrared spectroscopy, prefrontal function, twin study, verbal fluency task

## Abstract

**Objective:**

The genetic and environmental influences on prefrontal function in childhood are underinvestigated due to the difficulty of measuring prefrontal function in young subjects, for which near‐infrared spectroscopy (NIRS) is a suitable functional neuroimaging technique that facilitates the easy and noninvasive measurement of blood oxygenation in the superficial cerebral cortices.

**Method:**

Using a two‐channel NIRS arrangement, we measured changes in bilateral prefrontal blood oxygenation during a category version of the verbal fluency task (VFT) in 27 monozygotic twin pairs and 12 same‐sex dizygotic twin pairs ages 5–17 years. We also assessed the participant's full‐scale intelligence quotient (FIQ) and retrieved parental socioeconomic status (SES). Classical structured equation modeling was used to estimate the heritability.

**Results:**

The heritability of VFT‐related brain activation was estimated to be 44% and 37% in the right and left prefrontal regions, respectively. We also identified a significant genetic contribution (74%) to FIQ, but did not to VFT task performance. Parental SES was not correlated with FIQ, task performance, or task‐related prefrontal activation.

**Conclusions:**

This finding provides further evidence that variance in prefrontal function has a genetic component since childhood and highlights brain function, as measured by NIRS, as a promising candidate for endophenotyping neurodevelopmental disorders.

## 1 INTRODUCTION

The abilities to regulate thoughts and behaviors differ among people, and these differences have been investigated from the perspective of executive functions, a set of general‐purpose ability to control one's own cognition and actions to achieve higher goals (Miyake & Friedman, 2012).

Previous twin studies have revealed that a large part of interpersonal variance in executive function is explained by genetic components (Friedman et al., 2008; Polderman et al., 2006). In addition, environmental factors, such as parental socioeconomic status (SES), affect neurocognitive development, and children reared by parents with higher SES score are better in the executive function test compared with those reared by parents with lower SES (Hackman, Farah, & Meaney, 2010; Sarsour et al., 2011).

Twin studies that used functional magnetic resonance imaging (fMRI) have reported that individual differences in prefrontal brain activity associated with executive tasks, such as digit memory task and N‐back task, were partly explained by genetic factors (Blokland et al., 2008, 2011; Koten et al., 2009). In addition, polymorphisms in genes that are related to monoamine systems, such as catechol‐O‐methyltransferase, dopamine transporter, and serotonin transporter genes, were found to be associated with scores in executive tasks and prefrontal functions, as measured by fMRI (Barnes, Dean, Nandam, O'Connell, & Bellgrove, 2011). Those polymorphisms were suggested to have different effects in different age‐groups (Dumontheil et al., 2011).

Near‐infrared spectroscopy (NIRS) is a functional neuroimaging technique that facilitates the easy and noninvasive measurement of changes in blood oxygenation in the superficial cerebral cortices, which enabled its clinical application in psychiatry (Takizawa et al., 2014). Abnormalities in task‐related brain activation on NIRS have been detected in individuals with developmental disorders, such as attention deficit/hyperactivity disorder (Ehlis, Bähne, Jacob, Herrmann, & Fallgatter, 2008) and autism spectrum disorder (Kuwabara et al., 2006). NIRS studies also found similar abnormalities among children with developmental disorders and their unaffected siblings, which suggest a genetic influence on the changes in brain activation (Inoue et al., 2012; Kawakubo et al., 2009; Monden et al., 2015).

To further investigate the relationship between individual variance in brain functions and vulnerability to psychiatric disorders, it is vital to investigate how individual differences in brain function are attributable to genetic and environmental components. A previous study indicated that prefrontal activation during the executive task had significant heritability in healthy adults (Sakakibara et al., 2014). However, genetic and environmental influences on prefrontal function, as measured by NIRS, were not well investigated in children.

In this study, we investigated the heritability of prefrontal activation, as measured by NIRS, using a conventional twin study paradigm in children. We used a verbal fluency task (VFT) as the cognitive activation task because the VFT is a well‐established prefrontal task involving multiple domains of executive function, including self‐initiated retrieval of words from long‐term memory, working memory capacity to keep a track of the aforementioned items, and inhibition of inappropriate response (Henry & Crawford, 2004). Previous NIRS studies have reported that VFT stably activated prefrontal regions in both adults and children (Herrmann, Ehlis, & Fallgatter, 2003; Kawakubo et al., 2011). We hypothesized that the individual variance in task performance and task‐related brain activation, as measured by NIRS, would be partially explained by genetic factors. In addition, we expected that those measures would also be correlated with parental SES, a well‐established variable reflecting the richness of educational environment.

## 2 MATERIALS AND METHODS

### 2.1 Participants

Thirty‐nine same‐sex twins (78 participants) between the ages of 5 and 17 years were recruited via newspaper advertisements. Following a thorough explanation of the study requirements and procedures, all the participants provided informed assent, and parents or persons in parental authority of the participants provided written informed consent in accordance with the Declaration of Helsinki. All twin pairs had been reared together and were native Japanese speakers. Children with a history of psychiatric disorders were excluded using the Mini‐International Neuropsychiatric Interview (MINI) or MINI for children and adolescents (MINI‐KID) (Otsubo et al., 2005; Sheehan, Shytle, Milo, Janavs, & Lecrubier, 2009; Sheehan et al., 1998). Individuals with history of a neurological disorder, traumatic brain injury with loss of consciousness for more than 5 min, or a family history of any axis‐I disorder in a first‐degree relative were also excluded. The zygosity of twin pairs was determined using a three‐question questionnaire with more than 90% accuracy for diagnosing zygosity (Ooki, Yamada, Asaka, & Hayakawa, 1990). Twenty‐seven pairs were monozygotic (MZ) (11 male and 16 female pairs, 11.9 ± 3.5 years) and the remaining 12 pairs were dizygotic (DZ) (four male and eight female pairs, 10 ± 3.9 years). Parental SES was assessed using the Hollingshead scale in all but two families (Hollingshead, 1975). The average parental SES was 2.27 (range: 1–3), indicating that all families were in the middle or high SES groups. All participants completed the Japanese version of Wechsler Intelligence Scale for Children, third edition (WISC‐III) or the Wechsler Adult Intelligence Scale, revised (WAIS‐R) and full‐scale intelligent quotients (FIQ) were estimated. This study was approved by the Research Ethics Committee of the University of Tokyo Hospital (approval no. 630 and 2450).

### 2.2 Activation task

**Figure 1:**
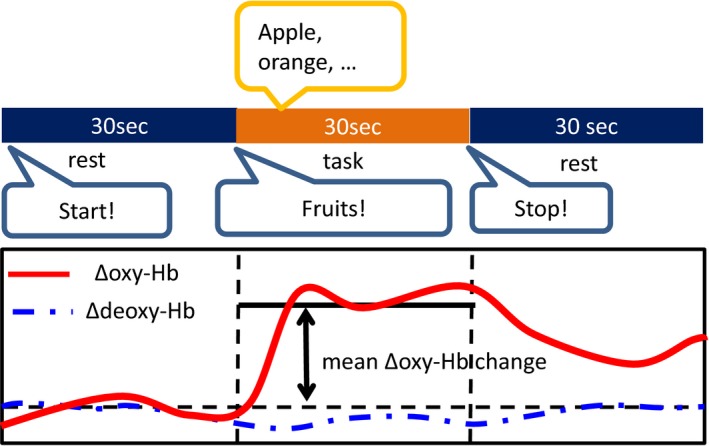
Design of the verbal fluency task (VFT). The VFT comprises a 30‐s rest period, a 30‐s task, and another 30‐s rest period. During the task period, participants were asked to name as many fruits as possible. Baseline correction was made by subtracting the average signal values during the 30‐s pretask resting period, and average Δoxy‐Hb values during the 30‐s task period were calculated for each hemisphere and defined as the magnitude of task‐related brain activation

Participants were seated on a chair with their eyes open and hands in their lap. The procedure for cognitive activation was adapted from previous studies (Kawakubo et al., 2009, 2011) and included a 30‐s rest period, a 30‐s verbal fluency task (category version), and another 30‐s rest period (Figure 1). During the task period, participants were asked to say as many names of fruits as possible. The auditory cue of “fruit” was presented at the start of the task period. Auditory cues were also presented at the beginning of each rest period. The number of correct words generated was used as a measure of task performance.

### 2.3 NIRS measurements

**Figure 2:**
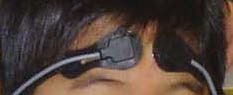
Placement of near‐infrared spectroscopy (NIRS) probes. The probes of a two‐channel NIRS apparatus (NIRO200, Hamamatsu Photonics, Inc.) were placed bilaterally on the subject's forehead to enable the measurement of Δoxy‐Hb and Δdeoxy‐Hb in the bilateral frontopolar prefrontal cortex

The NIRS machine and probe arrangement were identical to those used in a previous study (Kawakubo et al., 2009, 2011). Relative changes in oxygenated (Δoxy‐Hb) and deoxygenated (Δdeoxy‐Hb) hemoglobin signals were measured during the activation task using a two‐channel NIRS arrangement (NIRO200, Hamamatsu Photonics, Inc.) at three wavelengths of near‐infrared light (775, 810, and 850 nm). Each of the two probes included an emitter and a detector that were distanced by 4 cm. The probes were placed bilaterally on the subject's forehead using double‐sided adhesive tape so that the detectors were positioned at Fp1 and Fp2 with the emitters positioned 4 cm lateral to the detectors along the T3‐T4 line as per the international 10/20 system (Figure 2). We used the virtual registration method to locate the measurement positions in the cerebral cortex using a previously published method (Tsuzuki et al., 2007). The locations of NIRO probes and measurement areas were probabilistically estimated using independent adult magnetic resonance imaging data and anatomically labeled in the standard brain space (Brodmann's area). The estimated measuring areas of the right and left probes mostly corresponded to the right and left Brodmann's area 10 (frontopolar prefrontal cortex) (Kawakubo et al., 2011; Okamoto et al., 2004). We analyzed both Δoxy‐Hb and Δdeoxy‐Hb signals. However, we focused on Δoxy‐Hb because Δoxy‐Hb signals are more strongly correlated with blood oxygenation level‐dependent signal measured by fMRI than are Δdeoxy‐Hb changes (Strangman, Culver, Thompson, & Boas, 2002). Data were collected at a sampling rate of 0.5 s. Baseline correction was performed by subtracting the average signal values during the first 30 s of the resting period, and average Hb values during the 30‐s task period were calculated for each hemisphere and defined as the magnitude of task‐related brain activation. Data containing artifacts were removed by visual inspection. Only twin pairs

with complete data available were included in the analysis. As a result, 23 MZ pairs and nine DZ pairs were included in the analysis of left prefrontal activation, and 23 MZ pairs and 10 DZ pairs were included in the analysis of right prefrontal activation.

### 2.4 Statistical analysis

Twins were assigned a number 1 or 2 in accordance with birth order as indicated in their maternal and child health handbooks (the official birth record in Japan).

First, we tested the equality of means and homoscedasticity of demographic variables and task‐related brain activation across 4 groups (MZ twin1, MZ twin2, DZ twin1, and DZ twin2), which is the presupposition of genetic modeling, using a one‐way analysis of variance (ANOVA) and Levene's test. Correlations among MZ pairs and DZ pairs were calculated to compare similarity between MZ cotwins and DZ cotwins.

Genetic modeling was performed in accordance with classical structured equation modeling in twin studies (Neale & Cardon, 1992). An observed phenotypic value P was decomposed into a linear sum of an underlying additive genetic component (A), a genetic dominance component (D), a common environmental component (C), and a unique environmental component (E). Because the contributions of common environmental and genetic dominance cannot be estimated at the same time when data are unavailable from twins who were reared separately, we decided to adopt models comprising A, C, and E components (the ACE model). This model may underestimate but will not overestimate overall genetic contributions.

The full ACE model and its nested submodels (i.e., AE model, CE model, and E model) were compared to identify the most efficient model according to the Akaike information criterion, with the lowest value indicating the most efficient model. Parameters calculated from the most efficient model were considered to be final estimates. For each parameter, 95% confidence intervals were estimated using the bias‐corrected percentile method with 1000 cycles of bootstrapping.

It is known that age and sex differences affect the magnitude of task‐related brain activation (Kameyama, Fukuda, Uehara, & Mikuni, 2004). Because same‐sex cotwins are identical in age and sex, age‐ and sex‐related variance in task‐related activation may have induced the overestimation of common environmental components. In addition, previous studies found that cognitive functions are correlated with parental SES, and their heritability is lower in low SES families compared with high SES families (Turkheimer, Haley, Waldron, D'Onofrio, & Gottesman, 2003). Therefore, we tested if the measured variables (FIQ, task performance, and task‐related brain activation) were correlated with age, sex, and parental SES, and if correlated, we applied structured equation modeling to the residual values after removing the effects of those correlated variables to test the robustness of the results.

Additionally, we compared the similarity of waveforms between cotwins using Pearson's correlations. First, raw signals were moving averaged with a window of 5 s to remove heartbeat‐ and respiration‐related physiological signals. The coefficient of correlation r was then calculated for each twin pair and hemisphere as follows:

$$r=\frac{\sum_{t=1}^{180}\left(x_{1}\left(t\right)-{\overset{¯}{x}}_{1}\right)\left(x_{2}\left(t\right)-{\overset{¯}{x}}_{2}\right)}{\sqrt{\sum_{t=1}^{180}{\left(x_{2}\left(t\right)-{\overset{¯}{x}}_{2}\right)}^{2}}\sqrt{\sum_{t=1}^{180}{\left(x_{1}\left(t\right)-{\overset{¯}{x}}_{1}\right)}^{2}}}$$

where *x*_1_ (t) and *x*_2_ (t) denote the moving averaged signal intensity at time point *t* in twin 1 and 2, and $\overset{¯}{x}$ denotes the average signal intensity over time. Then, Pearson's correlation coefficients were converted to z scores using Fisher's z transformation. To test whether the z‐transformed coefficients among MZ twins were significantly different from those among DZ twins, we performed unpaired two‐sample t tests for each hemisphere. Statistical analyses were performed using the SPSS Amos version 20.0 software package (IBM Corp.) and MATLAB version R2013b (MathWorks, Inc.). For all analyses, *p *< .05 was the threshold for statistical significance.

## 3 RESULTS

### 3.1 Task performance and FIQ

There were no significant differences in task performance (*F*_3, 74_ = 0.327, *p *= .81) or FIQ (*F*_3, 74_ = 1.042, *p *= .38) among the four study groups (MZ twin1, MZ twin2, DZ twin1, and DZ twin2). Homoscedasticity was also maintained in these variables (*p *= .18 and 0.71, respectively).

Task performance and FIQ were moderately correlated between MZ cotwins and between DZ cotwins. The estimated coefficient for task performance was smaller in MZ cotwins than in DZ cotwins (0.441 vs. 0.726, respectively), whereas that for FIQ was larger in MZ cotwins than in DZ cotwins (0.707 vs. 0.444, respectively); however, these differences were not statistically significant. We fitted the full univariate ACE model and its nested submodels to task performance and FIQ. As a result, the most efficient model for task performance was the CE model and that for FIQ was the AE model (Table 1). Common environmental contribution was 0.56 (95% CI 0.30–0.75) for task performance, whereas the estimated heritability of FIQ was 0.74 (95% CI 0.51–0.88). The remaining variances (0.44 and 0.26, respectively) were attributed to unique environmental components.

**Table 1:** Correlation of cognitive and brain functions between cotwins and the estimates from the ACE model

	Means (SD)		Correlations (95% CIs)		A, C, and E (%) etimates (95% CIs)						
	MZ (*N *= 54)	DZ (*N *= 24)	MZ	DZ	Best model	X^2^	d.f.	*p*	A	C	E
Task performance	9.7 (3.1)	9.6 (4.1)	0.44 (0.07, 0.70)	0.73 (0.26, 0.92)	CE	3.29	4	.51		56 (30, 75)	44 (25, 70)
FIQ^a^	99.6 (12.4)	102.0 (15.8)	0.71 (0.45, 0.86)	0.44 (−0.17, 0.81)	AE	0.91	4	.92	74 (51, 88)		26 (12, 49)
Oxy‐Hb right prefrontal	0.49 (0.82)	0.09 (0.59)	0.52 (0.14, 0.77)	−0.45 (−0.84, 0.25)	AE	8.65	4	.07	44 (8, 70)		56 (30, 92)
Oxy‐Hb left prefrontal	0.52 (0.87)	0.10 (0.90)	0.37 (−0.05, 0.68)	0.27 (−0.48, 0.79)	AE	4.81	4	.31	37 (3, 69)		63 (31, 97)
Deoxy‐Hb right prefrontal	−0.05 (0.45)	−0.12 (0.36)	0.16 (−0.27, 0.54)	0.30 (−0.40, 0.78)	E	3.45	5	.63	0		100
Deoxy‐Hb left prefrontal	−0.07 (0.39)	−0.02 (0.24)	0.21 (−0.22, 0.58)	−0.11 (−0.72, 0.60)	E	6.53	5	.26	0		100

FIQ, full‐scale intelligent quotient; MZ, monozygotic twins; DZ, dizygotic twins; A, additive genetic components; C, common environmental components; E, unique environmental components.

a For participants aged 15 and under FIQ was evaluated with the WISC‐III, for participants aged 16 and over it was estimated by four subtests of the WAIS‐R.

Age was positively correlated with task performance (*r *= .702, *p *< .001), but not correlated with FIQ (*r *= .077, *p *= .64), as expected from the standardization by age. There was no significant sex difference for task performance or FIQ (unpaired t test, *p *= .28 and .61, respectively). Parental SES was uncorrelated with task performance (*r *= .094, *p *= .58) or FIQ (*r *= −.070, *p *= .68). We applied structured equation modeling to the residual values of task performance after removing the effects of age (linear and quadratic) using a multiple regression analysis, which made the E model most efficient for task performance.

### 3.2 NIRS results

**Figure 3:**
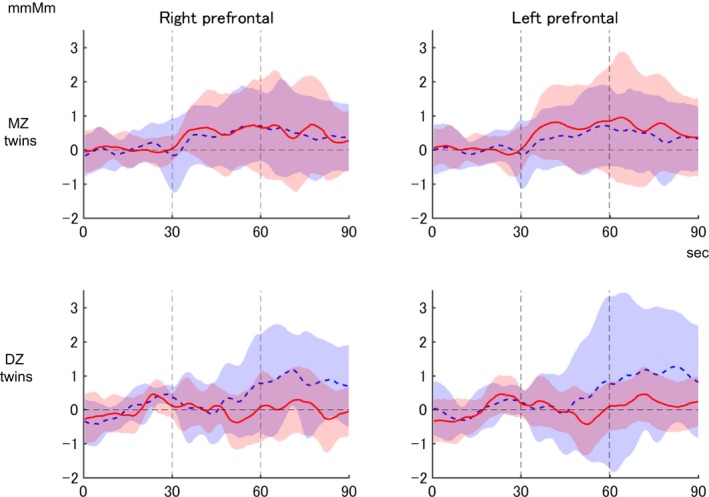
Grand average Δoxy‐Hb waveforms in monozygotic (MZ) and dizygotic (DZ) twins. Moving averaged Δoxy‐Hb fluctuations in the right and left prefrontal regions during the verbal fluency task are shown as grand average waveforms (twins 1 and 2 as red and blue lines, respectively) and their standard deviations (twins 1 and 2 as pink and light blue bands, respectively)

Average waveforms of Δoxy‐Hb during cognitive tasks are shown in Figure 3. There were no significant differences in task‐related brain activation in the right prefrontal (*F*_3, 62_ = 1.599, *p *= .20) or left prefrontal (*F*_3,60_ = 1.537, *p *= .21) regions among the four groups. Homoscedasticity was also maintained in these variables (*p *= .15 and .30, respectively).

**Figure 4:**
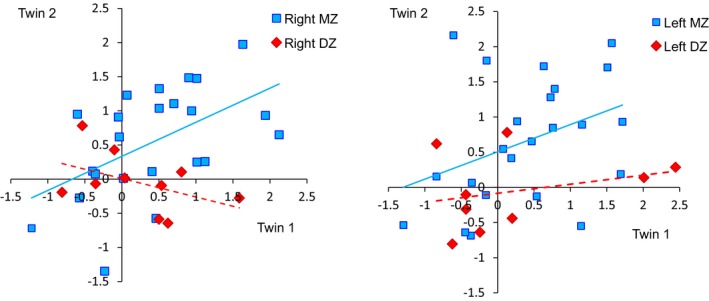
Scatter plot of task‐related brain activation in the right and left prefrontal regions . Blue squares represent monozygotic (MZ) twins and red diamonds represent dizygotic (DZ) twins. Blue solid and red dashed lines are linear regression lines of MZ and DZ data, respectively

Figure 4 shows a scatter plot of VFT‐related mean Δoxy‐Hb changes in each twin pair in the left and right prefrontal regions. As indicated in Table 1, correlations were only significant (positive) for right prefrontal activation between MZ cotwins. The estimated coefficient for right prefrontal activation between DZ cotwins was negative, but nonsignificant. The correlation of task‐related brain activation was larger between MZ cotwins than between DZ cotwins in both the right and left frontal regions. The AE model was the most efficient model for task‐related activation in both the right and left prefrontal regions. The estimated heritability values were 0.44 (95% CI 0.08–0.70) and 0.37 (95% CI 0.03–0.69), respectively.

Figure S1 shows a scatter plot of VFT‐related mean Δdeoxy‐Hb changes in each twin pair in the left and right prefrontal regions. The Δdeoxy‐Hb waveforms were nearly flat throughout the task periods and posttask periods. As indicated in Table 1, correlations of prefrontal activation were significant neither between MZ nor DZ cotwins, which made the E model the most efficient for both right and left prefrontal regions. Therefore, we performed the following additional analyses only to Δoxy‐Hb signals.

For Δoxy‐Hb, age was positively correlated with the magnitude of task‐related brain activation in both the right (*r *= .345, *p *= .03) and left (*r *= .552, *p *< .001) prefrontal regions. There was no sex difference in the magnitude of task‐related brain activation in the right or left prefrontal regions (unpaired t test, *p *= .13 and .13), although there was a trend of larger activation in female participants. Parental SES was uncorrelated with the magnitude of task‐related brain activation in the right (*r *= −.167, *p *= .32) or left (*r *= .004, *p *= .98) prefrontal regions. We applied structured equation modeling to the residual values after removing the effects of age (linear and quadratic) using a multiple regression analysis. As a result, the AE model remained the most efficient model for the right prefrontal region, whereas the E model was superior to the AE model for left prefrontal region. The estimate of heritability of task‐related brain activation in the right prefrontal region was reduced to 0.36 (95% CI 0.0–0.65, *p *= .03).

In the right prefrontal region, the mean correlation of waveforms between cotwins was significantly higher in MZ twins than in DZ twins in both the right (0.293 vs. −0.033; *p *= .009) and left (0.288 vs. 0.051; *p *= .16) prefrontal regions, although this effect was not significant for the left region.

## 4 DISCUSSION

The present twin study suggested bilateral prefrontal brain activation measured by NIRS during a VFT was partially determined by genetic factors from childhood. For the right prefrontal region, the effect remained significant after accounting for age. We also identified a significant genetic contribution to FIQ and a moderate common environmental contribution to task performance in the VFT (i.e., number of words generated), the latter of which disappeared after the variation of age was controlled. Finally, parental SES was not correlated with IQ, task performance, or task‐related prefrontal activation.

### 4.1 Heritability of prefrontal function and task performance

A previous fMRI study revealed the genetic influences on the activation of brain regions including the inferior frontal gyrus and anterior cingulate cortex during a digit memory task among male adults (Koten et al., 2009). Another group used an N‐back task to identify 40–65% heritability for task‐related activation in brain regions including the bilateral inferior, middle, and superior frontal gyri (Blokland et al., 2008, 2011). These findings are further supported by recent resting‐state functional MRI studies in twins. One such study of twins ages 12–19 years found that the amplitude of synchronous fluctuation in a small part of the right inferior gyrus constituting the executive control network was genetically determined, although the genetic influence was much stronger for sensory networks such as the visual, sensorimotor, and basal ganglia networks (Fu et al., 2015). A study of adult twins showed that the magnitude of synchronous fluctuation in the dorsal attention and frontoparietal networks in the prefrontal cortices showed 33% and 65% heritability, respectively (Yang et al., 2016). Furthermore, an adult twin NIRS study using the VFT as a cognitive task found that brain activation in the right dorsolateral prefrontal cortex and left frontal pole was genetically influenced, with 66% and 75% heritability, respectively (Sakakibara et al., 2014). The present finding is consistent with these previous results and provides further evidence that genetic control over prefrontal function begins in childhood.

In contrast, the heritability of task performance was not detected. Previous studies have demonstrated that executive functions, including verbal fluency, were highly heritable (Friedman et al., 2008; Hoekstra, Bartels, van Leeuwen, & Boomsma, 2009; Polderman et al., 2006). Heritability was also indicated for task performance in the aforementioned adult twin NIRS study (Sakakibara et al., 2014). The inconsistency may be because of the fact that task performance was strongly correlated with age (*r *= .702), which caused the overestimation of common environmental influence and complicated the detection of genetic influence.

### 4.2 Influence of parental SES

It is well established that parental SES is correlated with IQ and executive functions (Hackman et al., 2010). In addition, the heritability of IQ differs with parental SES, with children from higher SES families showing a higher heritability of IQ (Turkheimer et al., 2003). However, in our study, parental SES was not correlated with IQ, task performance, or task‐related prefrontal activation. The difference may be because of smaller variations in SES in our sample. Previous studies have considered both very low (below the poverty line) and high SES participants (Sarsour et al., 2011; Turkheimer et al., 2003); however, all the participants in our study were from middle or high SES families. It is expected from the fact that we recruited twins via newspaper advertisements and only parents who routinely read newspapers and were interested in academic research participated.

### 4.3 Brain function as a potential endophenotype of neurodevelopmental disorders

It is well established that the manifestation of psychiatric disorders is genetically influenced (Shimada‐Sugimoto, Otowa, & Hettema, 2015; Sullivan, Daly, & O'Donovan, 2012). Yet, the genetic architectures of many psychiatric disorders have yet to be identified. The identification of endophenotypes, namely biomarkers associated with specific psychiatric disorders and heritability, is a reasonable strategy for elucidating the complicated pathways relating genotypes and phenotypes (Gottesman & Gould, 2003). The present study suggests that prefrontal function during the VFT was subject to genetic influences as early as childhood. This finding indicates that brain function as measured by NIRS is a promising candidate of endophenotyping of neurodevelopmental disorders in children.

### 4.4 Limitations

The present study had several limitations. First, the number of participants in our study was smaller than that typical in behavior genetic studies, which usually involve several hundreds of participants. In addition, the distribution of participants in our study was unbalanced because there were more MZ pairs than DZ pairs, as well as more female pairs than male pairs. Therefore, the present results are preliminary, and we should refrain from inferring strong conclusions about the heritability of child brain function, as measured by NIRS.

Second, although the age range of participants in our study was wide (5–17 years), we were not able to test whether the heritability estimates varied with age due to the small sample size. It is indicated that the heritability of executive function differs with age (Hoekstra et al., 2009). Therefore, our results might be underestimating the heritability of prefrontal function for some age‐groups whereas overestimating others.

Third, the waveform during VFT showed significant task‐unrelated fluctuations, indicating the presence of substantial measurement errors. In the twin model, measurement errors are counted as unique environmental factors. Therefore, the mixing of measurement errors may underestimate genetic and common environmental factors. This may be the reason that our estimations of heritability (0.44 and 0.37) were smaller than those estimated among adults (66% and 75%) in a previous study (Sakakibara et al., 2014). Future studies should replicate our findings in larger and more balanced samples.

Fourth, brain activation estimated by NIRS is the product of changes in hemoglobin concentration and differential path length factor (DPF), the latter of which varies in accordance with individual brain structure. It is well established that brain structure is genetically influenced in childhood (Jansen, Mous, White, Posthuma, & Polderman, 2015). Therefore, estimated genetic influences on VFT‐related oxygenation changes in the prefrontal region may be partly confounded by the heritability of brain structure. When we compared the similarity of waveforms between cotwins using Pearson's correlations, the mean correlation of the waveforms was stronger between MZ cotwins than between DZ twins in the right prefrontal region. Because Pearson's correlation is unaffected by absolute signal values, the correlation coefficient between waveforms does not alter if the DPF varies between individuals. Therefore, our finding constitutes supportive evidence for a genetic influence on brain function.

## 5 CONCLUSION

We investigated the heritability of VFT‐related prefrontal activation as measured by NIRS using a conventional twin study paradigm. Whereas the heritability was undetected in VFT task performance, task‐related brain activation in right and left prefrontal regions showed a sign of genetic influences. Combined with the findings of previous twin studies using functional MRI and NIRS, the present result suggests that the variance in prefrontal function has a genetic component since childhood and that brain functions, as measured using NIRS, is a promising candidate for endophenotyping neurodevelopmental disorders in childhood.
