# Supplementary figures and images for: Genetic influences on prefrontal activation during a verbal fluency task in children: A twin study using near‐infrared spectroscopy
Source: Brain Behav. 2018 Apr 24;8(6):e00980. doi: 10.1002/brb3.980 (PMC5991600; doi:10.1002/brb3.980)

## Slide 1
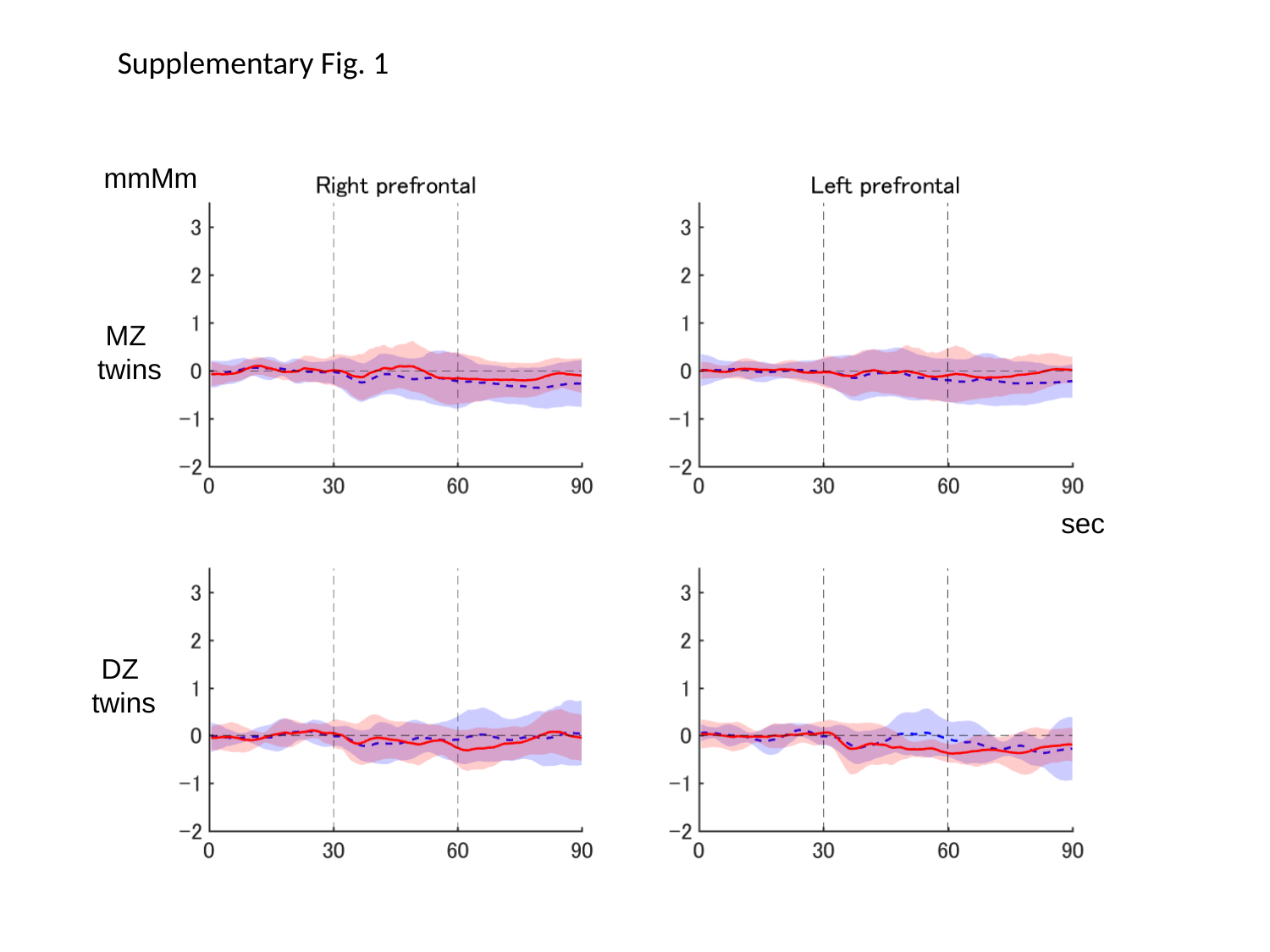

Supplementary Fig. 1
mmMm
MZ
 twins
sec
DZ
 twins

Supplement: Supplementary file 1 [file BRB3-8-e00980-s001.pptx]
